# Supplementary material for: A color image contrast enhancement method based on improved PSO
Source: PLoS One. 2023 Feb 9;18(2):e0274054. doi: 10.1371/journal.pone.0274054 (PMC9910741; doi:10.1371/journal.pone.0274054)
Supplement: Attachment — Submitted filename: Response to reviewers.docx [file pone.0274054.s005.docx]

List of Responses

Dear Editors and Reviewers，

On behalf of my co-authors, we thank you very much for giving us an opportunity to revise our manuscript, we appreciate editor and reviewers very much for their positive and constructive comments and suggestions on our manuscript entitled “A color image contrast enhancement method based on improved PSO”. (ID: PONE-D-21-36731).

Those comments are all valuable and very helpful for revising and improving our paper, and they are also having important guiding significance to us researches. We have studied those comments carefully and have made correction which are marked in red in the paper, hoping they will meet the standard. The main corrections and the responds to the reviewer’s comments are as flowing:

**Responds to the reviewer’s comments:**

**Reviewer #1:**

1. [Response to comment]:

The authors need to include the quality metrics in abstract to give a clear picture about quality of the proposed work.

[Responds]:

Considering the reviewer’s suggestion, we have written the quality metrics in abstract, clearly describing the quality of the proposed work.

2. [Response to comment]:

What is the novelty of the proposed PSO compared with existing PSO?

[Responds]:

In the manuscript, we have explained the proposed PSO in this part according to the reviewer’s suggestion. In order to ensure the diversity of particles, the PSO in this paper adds a local optimal value selected by the topology structure. And reduce the time complexity, a sparse penalty term is added to minimize the fitness value. The particles are penalized, and the effectiveness of the proposed PSO is verified by experiments.

3. [Response to comment]:

Need to separate introduction part and related work (literature survey part).

[Responds]:

Considering the reviewer’s suggestion, we have separated the introduction from the related work and added the related work of the literature survey.

4. [Response to comment]:

The author is expected to include a separate section for to discuss the metrics, PSNR SSIM IFC VIF FSIM.

[Responds]:

According to the reviewer’s suggestion, we have already introduced objective quality evaluation metrics in the experimental section.

5. [Response to comment]:

Measure the efficiency of the work using qualitative metrics such as Mean Opinion Score for reference kindly refer the following papers

[1] Swarm intelligent based contrast enhancement algorithm with improved visual perception for color images

[2] Particle swarm optimisation aided weighted averaging fusion strategy for CT and MRI medical images

[3] An optimal weighted averaging fusion strategy for remotely sensed images

[4] Optimal fusion aided face recognition from visible and thermal face images.

[Responds]:

In the experimental part, by referring to several of the above literatures, we have added a qualitative indicator (Mean Opinion Score, MOS) to measure the efficiency of the work.

**Reviewer #2:**

1. [Response to comment]:

The language and presentation need to proof-read and refined.

[Responds]:

We have revised the grammar carefully to make the paper smoother and more informative, which was done by us as well as the editing company.

2.[ Response to comment]:

There is no novelty in the transformation function, fitness function etc. It is the same as in Ref [1] The improved PSO algorithm is made use to optimize the selection of parameters k, a, b, c. Authors did not provide any clear mathematical, analytical or theoretical proof to substantiate how the improved version could perform much better as compared with conventional PSO.

[Responds]:

In the manuscript, we have analyzed the four parameters of the improved PSO optimization are better than the traditional PSO optimization.

3. [Response to comment]:

Comparisons with recent methods are missing. Include comparisons with few state-of-the art non-metaheuristic algorithms as well.

[Responds]:

Considering the reviewer’s suggestion, we have compared in this paper three recent image enhancement methods, but not based on meta-heuristics.

4. [Response to comment]:

Details about the dataset used is missing.

[Responds]:

In the manuscript, we have completed the details of the dataset.

5. [Response to comment]:

Since a deep layered structure was used in the improved variant of PSO proposed, quantify the computational complexity of the entire algorithm

[Responds]:

Considering the Reviewer’s suggestion, we have quantified the computational complexity of the proposed deep-structured particle swarm optimization.

6. [Response to comment]:

The deep layered structure of proposed algorithm is unclear. How are the parameters included in it tuned and selected? Are they image dependent?

[Responds]:

In the manuscript, we have detailed the deep topology, how the parameters are tuned and selected. They are not dependent on images.

7. [Response to comment]:

Since metaheuristic algorithms always give slightly different results in each run. Include statistical analysis as well. The results seem to be manipulated, since there is no chance to have such an improvement in the quantitative and qualitative results using the proposed approach. If not, substantiate clearly.

[Responds]:

Since metaheuristic algorithms always give slightly different results in each run. In this paper, the results are obtained after obtaining the average value through multiple experiments, and the experimental image is the optimal value obtained after optimization.

**Responds to the Editor’s comments:**

1. [Response to comment]:

You note that your data are available within the Supporting Information files, but no such files have been included with your submission. At this time, we ask that you please upload your minimal data set as a Supporting Information file, or to a public repository such as Figshare or Dryad.

Please also ensure that when you upload your file you include separate captions for your supplementary files at the end of your manuscript.

[Responds]:

We have uploaded the supporting information and added a separate title to the Supporting Information file at the end of the manuscript.

2. [Response to comment]:

We note that Figures 4 and 5 in your submission contain copyrighted images. All PLOS content is published under the Creative Commons Attribution License (CC BY 4.0), which means that the manuscript, images, and Supporting Information files will be freely available online, and any third party is permitted to access, download, copy, distribute, and use these materials in any way, even commercially, with proper attribution.

We require you to either (1) present written permission from the copyright holder to publish these figures specifically under the CC BY 4.0 license, or (2) remove the figures from your submission:

a) You may seek permission from the original copyright holder of Figures 4 and 5 to publish the content specifically under the CC BY 4.0 license.

b) If you are unable to obtain permission from the original copyright holder to publish these figures under the CC BY 4.0 license or if the copyright holder’s requirements are incompatible with the CC BY 4.0 license, please either i) remove the figure or ii) supply a replacement figure that complies with the CC BY 4.0 license. Please check copyright information on all replacement figures and update the figure caption with source information. If applicable, please specify in the figure caption text when a figure is similar but not identical to the original image and is therefore for illustrative purposes only.

[Responds]:

In the manuscript, the copyrighted images contained in Figures 4 and 5 in the submission have been removed and replaced with images from our homemade dataset, under the CC BY 4.0 license. All images are completely taken by myself and do not involve copyright information.

Thank you for your constructive comments.

We tried our best to improve the manuscript and made some changes in the manuscript. These changes will not influence the content and framework of the paper. We need to show our heartfelt appreciation for Editors/Reviewers’ efforts, and hope that the correction will meet with approval.

Once again, thank you very much for your comments and suggestions.

Yours sincerely,

Ren Yongfeng

E-mail: [503212590@qq.com](mailto:503212590@qq.com)
